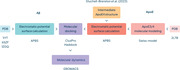# Interrogating the molecular interaction between apolipoprotein E and the beta‐amyloid peptide

**DOI:** 10.1002/alz.089469

**Published:** 2025-01-03

**Authors:** Mateus V Araujo, Mychael V Lourenco, Pedro H M Torres

**Affiliations:** ^1^ Institute of Biophysics Carlos Chagas Filho, Federal University of Rio de Janeiro (UFRJ), Rio de Janeiro, Rio de Janeiro Brazil; ^2^ Institute of Medical Biochemistry Leopoldo de Meis, Federal University of Rio de Janeiro, Rio De Janeiro, Rio de Janeiro Brazil

## Abstract

**Background:**

The apolipoprotein ε4 (ApoE4) allele is a major risk factor for sporadic Alzheimer’s disease (AD) and was shown to promote amyloid‐β (Aβ) accumulation and mediate pathophysiological processes in AD. Although the molecular interaction between Aβ and ApoE is acknowledged, the precise nature of this interaction remains unclear. This study aims to explore the biophysical and biochemical nature of the interaction between Aβ and ApoE in the ε3 and ε4 isoforms.

**Method:**

We initially reverted 5 point mutations to generate the original ApoE3 structure from its full‐length structure (PDB: 2L7B), by using the Swiss‐Model software. We then inserted the 112CtoR mutation to generate ApoE4. We further used a previously published open‐state structure of ApoE4. Three distinct Aβ42 structures (PDB: 1IYT, 6SZF, 1Z0Q) were used. Docking preparations were performed using ChimeraX with PDB2PQR and APBS used to generate electrostatic surfaces. ClusPro and Haddock software facilitated rigid and flexible molecular docking, respectively. This study performed nine dockings in total, selecting six suitable complexes for coarse‐grained molecular dynamics in triplicate using GROMACS.

**Result:**

The Aβ42 structure that emulates a membrane‐associated environment (1IYT) provided the best fits in the molecular docking, followed by the pre‐transition to β‐sheet structure (1Z0Q). The “intermediate structure” (6SZF), a stage between the two other structures, resulted in unspecific docking. The molecular dynamics from the promising structures are being carried out to understand the different behavior and stability at pH 7.4. We are also undertaking additional dynamics experiments to investigate the influence of an acidic pH (5.5).

**Conclusion:**

We initially confirmed the molecular interaction between Aβ42 and ApoE isoforms. This interaction is influenced by the local environment. Further results are expected to elucidate molecular details of this interaction hold potential to enhance our understanding of AD pathophysiology.